# Arsenic modifies the effect of folic acid in spina bifida prevention, a large hospital-based case-control study in Bangladesh

**DOI:** 10.1186/s12940-024-01091-1

**Published:** 2024-06-03

**Authors:** Chih-Fu Wei, Sudipta Kumer Mukherjee, Sheikh Muhammad Ekramullah, D. M. Arman, Md Joynul Islam, Mubinul Azim, Asifur Rahman, Md Nafaur Rahman, Md Ziauddin, Gwen Tindula, Hafiza Sultana Suchanda, Diana F. Gomberg, Marc G. Weisskopf, Liming Liang, Benjamin C. Warf, David C. Christiani, Maitreyi Mazumdar

**Affiliations:** 1 Department of Environmental Health, Harvard T.H. Chan School of Public Health, 655 Huntington Avenue, Boston, MA 02115 USA; 2 Department of Paediatric Neurosurgery, National Institute of Neurosciences & Hospital, Sher-e-Bangla Nagar, Agargoan, Dhaka 1207 Bangladesh; 3 Department of Clinical Neurosurgery, National Institute of Neurosciences & Hospital, Sher-e-Bangla Nagar, Agargoan, Dhaka 1207 Bangladesh; 4 Dhaka Shishu Hospital, Dhaka, Bangladesh; 5 Department of Neurosurgery, Bangabandhu Sheikh Mujib Medical University (BSMMU), Dhaka, Bangladesh; 6 Department of Epidemiology and Population Health, Stanford University, Palo Alto, , 300 Pasteur Drive, CA 94305 USA; 7 Pediatric Neurosurgery Research Committee, National Institute of Neurosciences & Hospital, Sher-e-Bangla Nagar, Agargoan, Dhaka 1207 Bangladesh; 8 Department of Neurology, Boston Children’s Hospital, BCH3443, 300 Longwood Ave, Boston, MA 02115 USA; 9 Department of Biostatistics, Harvard T.H. Chan School of Public Health, 655 Huntington Avenue, Boston, MA 02115 USA; 10 Department of Neurosurgery, Boston Children’s Hospital, 300 Longwood Avenue, Boston, MA 02115 USA

**Keywords:** Arsenic, Bangladesh, Folic acid, Spina bifida

## Abstract

**Background:**

Spina bifida, a developmental malformation of the spinal cord, is associated with high rates of mortality and disability. Although folic acid-based preventive strategies have been successful in reducing rates of spina bifida, some areas continue to be at higher risk because of chemical exposures. Bangladesh has high arsenic exposures through contaminated drinking water and high rates of spina bifida. This study examines the relationships between mother’s arsenic exposure, folic acid, and spina bifida risk in Bangladesh.

**Methods:**

We conducted a hospital-based case-control study at the National Institute of Neurosciences & Hospital (NINS&H) in Dhaka, Bangladesh, between December 2016 and December 2022. Cases were infants under age one year with spina bifida and further classified by a neurosurgeon and imaging. Controls were drawn from children seen at NINS&H and nearby Dhaka Shishu Hospital. Mothers reported folic acid use during pregnancy, and we assessed folate status with serum assays. Arsenic exposure was estimated in drinking water using graphite furnace atomic absorption spectrophotometry (GF-AAS) and in toenails using inductively coupled plasma mass spectrometry (ICP-MS). We used logistic regression to examine the associations between arsenic and spina bifida. We used stratified models to examine the associations between folic acid and spina bifida at different levels of arsenic exposure.

**Results:**

We evaluated data from 294 cases of spina bifida and 163 controls. We did not find a main effect of mother’s arsenic exposure on spina bifida risk. However, in stratified analyses, folic acid use was associated with lower odds of spina bifida (adjusted odds ratio [OR]: 0.50, 95% confidence interval [CI]: 0.25-1.00, *p* = 0.05) among women with toenail arsenic concentrations below the median value of 0.46 µg/g, and no association was seen among mothers with toenail arsenic concentrations higher than 0.46 µg/g (adjusted OR: 1.09, 95% CI: 0.52–2.29, *p* = 0.82).

**Conclusions:**

Mother’s arsenic exposure modified the protective association of folic acid with spina bifida. Increased surveillance and additional preventive strategies, such as folic acid fortification and reduction of arsenic, are needed in areas of high arsenic exposure.

**Supplementary Information:**

The online version contains supplementary material available at 10.1186/s12940-024-01091-1.

## Background

Spina bifida is a developmental malformation of the spinal cord that leads to increased risk of mortality and disability, including leg, bladder, and bowel dysfunction, susceptibility to infection, hydrocephalus, and cerebrospinal fluid (CSF) leakage [1]. Spina bifida is a type of neural tube defect (NTD), a group of disorders caused by failure of the neural tube to fuse in the third week of gestation [1]. A recent analysis estimated that globally, at least 213,800 − 322,000 pregnancies are affected by NTDs each year, and in low-income and middle-income countries, the prevalence of NTDs exceeds one in every 100 births [2].

Folic acid supplement use reduces the risk of spina bifida [3], and fortification of staple foods with folic acid has been successful in decreasing spina bifida rates in multiple countries [4, 5]. However, a substantial number of affected pregnancies occur in areas with folic acid fortification and to women known to have taken folic acid supplements [6], and the effectiveness of folic-acid based preventive strategies varies significantly across and even within countries [2, 5]. There is an urgent need to identify modifiable factors that may reduce the burden of this condition.

Environmental exposure to arsenic is of particular concern and may be a potential contributor to spina bifida risk, especially in areas of the world with high arsenic exposures and micronutrient deficiencies. Arsenic induces neural tube defects in several animal models [7–11], and arsenic toxicity is closely related to one-carbon metabolism nutrients including folate [12]. A recent systematic review showed there was inadequate evidence to determine the relationship between prenatal arsenic exposure and prevalence of NTDs [13], but the review did not address potential interactions between arsenic and folic acid that may affect spina bifida risk.

Understanding the relationship between folic acid, spina bifida, and arsenic exposure is especially important in Bangladesh, where an estimated 70 million people are chronically exposed to high concentrations of arsenic through contaminated groundwater, in what has been described as the largest mass poisoning in history [14, 15]. In 2019, a national survey conducted by the Bangladesh Bureau of Statistics and United Nations Children’s Fund estimated that 18.6% of households in Bangladesh were exposed to source water arsenic levels > 10 µg/L (the current World Health Organization drinking water standard for arsenic) and 11.8% were exposed to levels > 50 µg/L [16]. Bangladesh also has high rates of spina bifida. The country does not have systematic surveillance for birth defects, but hospital-based studies estimate the prevalence of spina bifida to be between 10.4 and 38.2 per 10,000 births, higher than the global prevalence of 3.5 to 5.2 per 10,000 in prior meta-analyses [5, 17, 18].

Our previous study discovered that higher water arsenic concentrations reduced the effectiveness of folic acid in spina bifida prevention [19]. Building on these results, we conducted a larger, hospital-based case-control study at the national center for spina bifida treatment in Bangladesh. We collected various biomarkers to investigate how arsenic contributes to the risk of spina bifida. For example, we use toenail arsenic as a biomarker. Previous reports from this population identified an association between father’s arsenic concentrations in fathers’ toenails and spina bifida [20]. In this study, we sought to ascertain whether mother’s arsenic exposure is associated with higher risk of spina bifida and whether arsenic exposure modified the protective effect of folic acid supplementation in Bangladesh.

## Methods

### Case ascertainment, and control selection

We conducted a hospital-based case-control study at the National Institute of Neurosciences & Hospital (NINS&H), the primary center for spina bifida surgery in Bangladesh. Cases were infants with spina bifida who were less than one year old and had mothers who could identify their primary drinking water source during early pregnancy. No exclusions were made based on genetics, geography, or family history. Neurosurgeons at NINS&H examined all cases and reviewed medical records, operative reports, and available imaging results. Study staff recorded the subtype of spina bifida (myelomeningocele or meningocele), level of lesion (e.g., cervical, thoracic, lumbar, sacral, or lumbosacral), co-occurring anomalies, and complications present at time of enrollment, including cerebrospinal fluid leak, lesion infection, and hydrocephalus. A senior neurosurgeon (BCW) confirmed classification of cases by reviewing photographs and available imaging.

We selected controls from children who presented to NINS&H or Dhaka Shishu Hospital (DSH), a children’s hospital nearby NINS&H with similar referral patterns and catchment areas. When a case was enrolled, we reviewed that week’s clinic lists and assembled a list of potential controls based on age within 6 months of the case and approached potential controls in order of the list. Eligibility criteria for cases are presented in Supplemental Table 1. We did not include children with cancer (e.g. brain tumor) as controls because the link between arsenic and cancer is established in the literature [21], and thus we believed presentation of children with cancer to NINS&H and DSH is not independent of arsenic exposure [22]. The Bangladesh Medical Research Council and the Human Research Committees at Boston Children’s Hospital (BCH), NINS&H, and DSH approved this study (BCH protocol number IRB-P00019768; BMRC registration number: 006 23 08 2016). The Harvard T.H. Chan School of Public Health ceded review to BCH (protocol number: IRB20-0780). Parents provided informed consent before enrollment.

### Clinical information and folate status

Trained study staff interviewed patients’ families to collect demographic characteristics, patient and family medical histories, referral patterns to NINS&H, and medical histories. We measured infant’s weight and head circumference using standardized protocols, and mother’s medication and vitamin intake using a structured questionnaire that included the seven major types of folic acid-containing tablets in Bangladesh. We also collected information about the timing of initiation of vitamin use (before or after knowing about pregnancy), duration and frequency. To estimate nutritional intake during pregnancy, we administered a food frequency questionnaire previously validated in Bangladesh [23]. Folate status was additionally assessed in serum samples using Chemiluminescent Microparticle Immunoassay at NINS&H (ARCHITECT plus ci4100, Abbott Company, Abbott Park, IL, USA).

### Water arsenic concentration

At enrollment, mothers were asked to identify the primary drinking water source they used when they learned they were pregnant. Trained staff visited these sites and collected water samples in polyethylene containers. Arsenic concentrations in water were assessed at the Bangladesh University of Engineering and Technology using graphite furnace atomic absorption spectrometry (GF-AAS) with a limit of detection (LOD) of 1 µg/L [24]. For water arsenic concentrations below the limit of detection (LOD), we assigned a value of LOD/$\sqrt{2 }.$ We tested one blank for every 50 water samples.

### Toenail arsenic concentration

Mother’s toenail clippings were obtained from all toenails and placed in a small coin envelope. The samples were stored and shipped to the Dartmouth Trace Element Analysis Core at room temperature. Arsenic concentrations were measured using inductively coupled plasma mass spectrometry (ICP-MS) using methods that have been previously described [25]. The instrument’s LOD was 0.01 ng/g for the first 338 samples, and 0.1 ng/g for the remaining samples. For toenail arsenic concentrations below the instrument’s LOD, we assigned a value equal to the average dilution factor of toenail samples multiplied by LOD/$\sqrt{2 }$.

### Statistical analysis

To compare the data between cases and controls, we used t-tests for continuous variables, and chi-square and Fisher’s exact tests for categorical variables. We calculated Spearman correlation coefficients for the correlations of toenail and water arsenic concentrations.

We employed logistic regression models to assess the associations between arsenic concentrations and folic acid use (predictors) and case status. We modeled arsenic concentrations in two ways: (1) as a continuous variable and (2) as a binary variable: above and below 10 µg/L for water, and above and below the median for toenails. We did not use conditional models because of the uneven numbers of cases and controls. Models were adjusted for potential confounders that were chosen based on prior knowledge and using directed acyclic graphs and included mother’s age, place of birth (hospital, clinic, or home) and secondhand smoke exposure. Because of increasing understanding that myelomeningocele and meningocele are genetically distinct disorders, we restricted the cases to only those with myelomeningocele in sensitivity analyses [26]. Data were analyzed using R (version 4.0.4).

## Results

We enrolled 333 infants with initial diagnoses of spina bifida (meningocele or myelomeningocele) and 165 controls. We stopped enrolling controls and collecting water in March 2020 because of COVID-19 restrictions, and this led to an uneven number of cases and controls. Participation rates were 73% among potential families with spina bifida and 57% among potential controls. Reasons for study refusal were similar among cases and controls and primarily included concerns about giving blood. We excluded 11 cases because surgery revealed lipomeningocele and 1 case because the mother reported valproic acid use. Toenail arsenic concentrations were not available for 27 cases and 2 controls. Our final study population included 294 cases and 163 controls. Diagnoses of the controls are presented in Supplemental Table 2.

Demographic information is presented in Table 1. Thirteen mothers reported a diagnosis of diabetes, including mothers of 9 cases and 4 controls. Prenatal folic acid use among our study population was low, but consistent with other reports from Bangladesh; [27–29] only 16.7% of mothers of cases in our study and 22.1% of mothers of controls reported using folic acid during pregnancy. Serum folate concentrations were higher among mothers who reported folic acid use during pregnancy compared to those who did not report folic acid use (10.20 ng/ml and 7.96 ng/ml, respectively). On average, arsenic concentrations in water were lower than seen in our previous studies in Bangladesh [19, 30], although some high arsenic concentrations were seen (maximum water arsenic concentration: 451 µg/L) (Table 2). We observed a moderate correlation between water and toenail arsenic concentrations (Spearman correlation coefficient: 0.45). Arsenic was detected in all toenail samples.

**Table 1:** Characteristics of infants with spina bifida and their mothers compared to controls

	Cases (*n* = 294)	Controls (*n* = 163)	*p*-value
***Infant characteristics***			
Boys	157 (53.4)	104 (63.8)	0.04
Firstborn	102 (34.7)	87 (53.4)	< 0.001
Gestational age (week)	37.3 (2.1)	37.0 (2.3)	0.22
Age at study visit (days)	75.5 (86.6)	185.3 (96.1)	< 0.001
Myelomeningocele	249 (84.7)	NA	
Level of spina bifida			
Cervical or thoracic	11 (3.7)	NA	
Lumbar	144 (49.0)	NA	
Sacral or lumbosacral	138 (46.9)	NA	
***Mother’s characteristics***			
Age (years)	24.7 (4.7)	24.3 (5.0)	0.39
Education level			0.11
Illiterate	3 (1.0)	2 (1.2)	
Literate	55 (18.7)	22 (13.5)	
High school or less	164 (55.8)	83 (50.9)	
College	56 (19.0)	38 (23.3)	
University	16 (5.4)	18 (11.0)	
Folic acid use during early pregnancy	49 (16.7)	36 (22.1)	0.19
Living in Dhaka	153 (52.0)	89 (54.6)	0.67
Pre-pregnancy diabetes	9 (3.1)	4 (2.5)	0.78
Secondhand smoke exposure	127 (43.2)	60 (36.8)	0.22
Fever episodes during pregnancy	153 (52.0)	70 (42.9)	0.06
Place of birth			0.16
Home	79 (26.9)	41 (25.2)	
Clinics	111 (37.8)	50 (30.7)	
Hospitals	104 (35.4)	72 (44.2)	

Mean (SD) or number (%); NA = not applicable

**Table 2:** Arsenic concentrations in toenails and drinking water

	Mean (SD)	Median	IQR	Min	Max
***Mother’s toenail arsenic concentration (µg/g)***					
Cases (*n* = 294)	1.04 (1.58)	0.45	0.28–1.05	0.07	13.77
Controls (*n* = 163)	1.13 (1.94)	0.47	0.30–0.96	0.09	12.30
***Water arsenic concentration (µg/L)***					
Cases (*n* = 166)	15.35 (37.28)	2.00	1.00–7.00	0.71	255.00
Controls (*n* = 161)	20.91 (56.08)	2.00	0.71-7.00	0.71	451.00

a. Water arsenic concentrations were below the LOD (1 µg/L) for 41 cases and 52 controls. Arsenic was detected in all toenail samples, and the LOD for toenail arsenic was 0.01 ng/g for first 338 samples tested and 0.1 ng/g for remaining samples

b. Water samples were not available for participants enrolled during the COVID-19 pandemic due to travel restrictions in Bangladesh.

Abbreviations: IQR, interquartile range; LOD, limit of detection; SD, standard deviation.

**Fig. 1:**
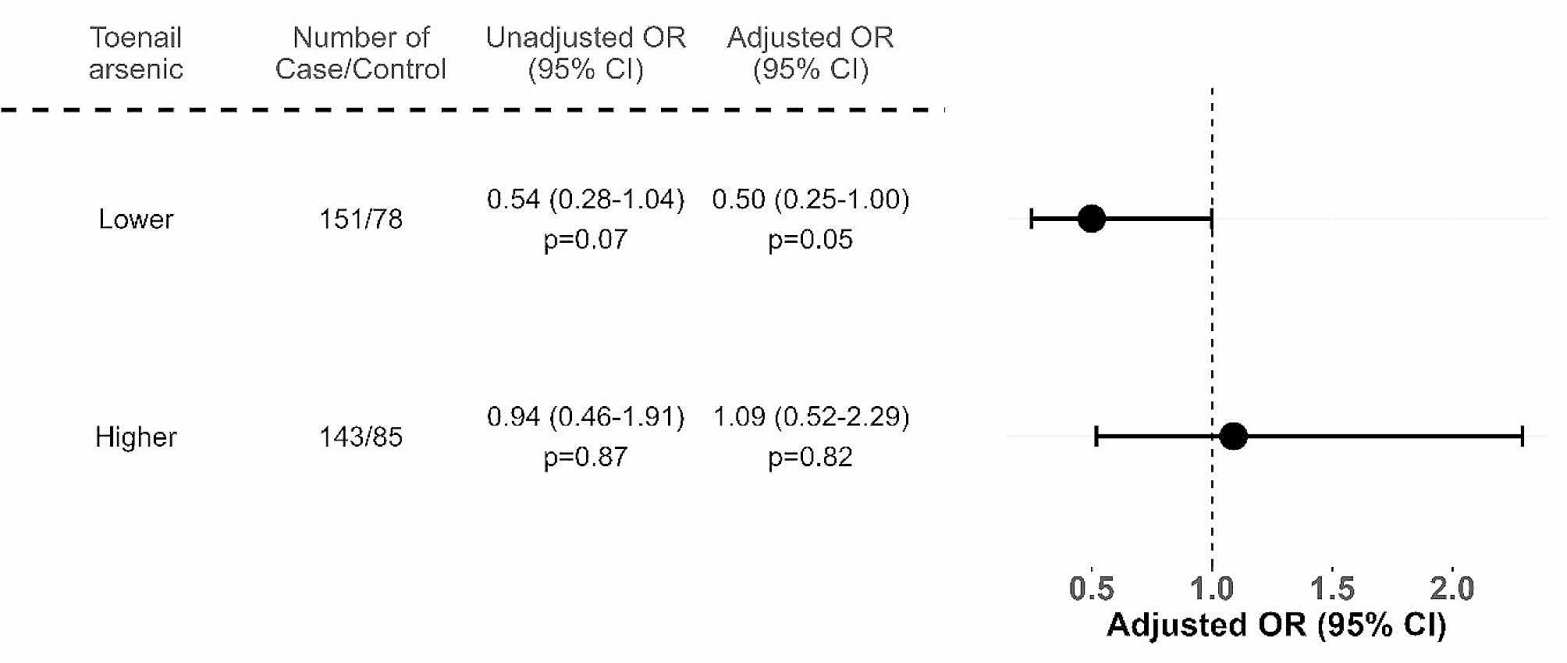
Association between prenatal folic acid use and spina bifida risk, by mother’s toenail arsenic concentration Adjusted for mother’s age (years), place of birth (hospital, clinic, or homes), and secondhand smoke exposure. The cutoff for mother’s toenail arsenic concentrations was defined by the median of study population (0.46 µg/g toenail). Abbreviations: CI, confidence interval; OR, odds ratio

There was no significant main effect found between water and mother’s toenail arsenic concentrations and spina bifida risk (Supplemental Table 3). However, in stratified models, we found evidence that arsenic exposure modified the effect of folic acid on spina bifida risk (Fig. 1). Among mothers with toenail arsenic concentrations below the median, folic acid use during pregnancy was associated with lower odds of having an infant with spina bifida (adjusted odds ratio [OR]: 0.50, 95% confidence interval [CI]: 0.25–1.00). Among women with toenail arsenic concentrations above the median, this protective association was not seen (adjusted OR: 1.09, 95% CI: 0.52–2.29). We found similar results after restricting the cases to those with myelomeningocele (Supplemental Fig. 1) (adjusted OR: 0.45, 95% CI: 0.21–0.94 vs. adjusted OR: 1.03, 95% CI: 0.48–2.23). When using water arsenic concentration as our measure of arsenic exposure, folic acid had a protective association at arsenic concentrations > 10 µg/L (adjusted OR: 0.18, 95% CI 0.04–0.93), but this finding may be limited by the small numbers of reported folic acid users in this group (Supplemental Fig. 2).

## Discussion

Our study found that arsenic modifies the effect of folic acid on spina bifida prevention in a population in Bangladesh, a country with high arsenic exposures through contaminated drinking water. Among mothers with toenail arsenic concentrations below the median, folic acid was associated with a protective effect (adjusted OR:0.50, 95% CI: 0.25-1.00), but a protective effect was not observed among mothers with toenail concentrations above the median (adjusted OR:1.09, 95% CI: 0.52–2.29). We found similar evidence of effect modification when we restricted the analysis to cases with myelomeningocele, a more severe type of spina bifida. (Supplemental Fig. 1)

Our results are consistent with recent studies conducted in high arsenic areas that suggest that environmental arsenic exposure may attenuate the protective benefit of folic acid supplements in preventing spina bifida. In a large case-control study of NTDs in Shanxi, China, researchers found that placental arsenic concentrations were associated with NTD-affected pregnancies among mothers who reported not taking folic acid supplements [31]. Our previous studies in Bangladesh found that as drinking water arsenic concentrations increased from 1 µg/L to 25 µg/L, the protective effect of folic acid use declined (OR: 0.22, 95% CI: 0.13, 0.37 to OR: 1.03, 95% CI: 0.55, 1.91) [19]. These studies suggest that prenatal folic acid supplementation may be less effective in spina bifida prevention in populations with high arsenic exposure because of interactions between arsenic and folic acid.

Animal studies have consistently shown arsenic to be a potent teratogen, inducing neural tube defects in several animal models [7–11]. In addition, animal studies demonstrate that arsenic-folate interactions are important in arsenic’s teratogenicity. In *Folbp2* knockout mice, for example, mice with this specific defect in folate transport had higher rates of NTDs after arsenic exposure, and mice nullizygous for genes encoding proteins in cellular uptake of folate were more susceptible to arsenic-induced NTDs [32]. The interaction between arsenic and folate may be explained by arsenic’s interference with folate-related functions, such as the depletion of S-adenosylmethionine (SAM), a key methyl donor for pathways implicated in neural tube closure [12]. In experimental models, chicken embryos exposed to 100nM arsenate showed reduced SAM levels and higher rates of NTDs [33]. In humans, the strongest evidence for arsenic-folic acid interactions comes from trials in which folic acid has been shown to reduce blood arsenic concentrations [34–36]. 

Although we found evidence of effect modification by arsenic, our study did not demonstrate a primary (or main) effect of mother’s arsenic exposure on spina bifida risk, consistent with findings from a recent systematic review assessing cohort studies that had measures of prenatal arsenic exposure and spina bifida outcomes [13]. It is possible that even higher arsenic exposures are needed to demonstrate a primary effect. A recent study from an area of Turkey with high arsenic exposures reported higher levels of arsenic in plasma samples of 100 mothers of NTD cases as compared to mothers from 70 controls [37]. Survivorship bias may also account for not finding a primary effect. We enrolled cases at time of presentation for surgical care and did not capture affected pregnancies that did not continue to birth or more severely affected infants who were not brought for care. If arsenic exposure was related to these more severe outcomes, our results could represent a downward bias towards or beyond the null [38]. Another possible explanation for why we did not find a primary effect of arsenic is that arsenic exposure by itself may be insufficient, and that the addition of other risk factors, such as inadequate folate, is a necessary contribution to disrupt neural tube closure.

There were two main limitations to the study. First, recall bias may arise since information about folic acid use and drinking water sources was collected after occurrence of spina bifida and might be influenced by differential recall between mothers of cases and mothers of controls. We used biomarkers (e.g. toenail arsenic) to minimize this bias. Second, survivorship bias could affect results. We were not able to assess all pregnancies that included neural tube defects because Bangladesh does not have a birth defect surveillance program that collects this information from ultrasonography centers or from all deliveries. If arsenic is associated with pregnancy loss or stillbirth due to neural tube defects, our study would underestimate the association between arsenic and spina bifida because it did not capture the most severely affected cases.

The strengths of our study include the use of individual measures of exposure, including toenail arsenic measurements, which represent arsenic exposure from 5 to 18 months before collection [39], and may better represent exposure during early pregnancy than blood, urine, or water samples collected after delivery. Previous work in Bangladesh in a large pregnancy cohort has shown high correlations between pregnant women’s toenail arsenic measurements during the first trimester and 1 month post-partum and also with measurements from their infant’s toenails at age 1 month [30]. An additional strength is classification of cases by neurosurgeons using examination, imaging and observations during surgery. It is increasingly understood that NTDs are not one disorder, but instead a wide array of morphologically distinct malformations which likely each result from a different contribution of risk factors [40]. Our study is the largest study to date to investigate spina bifida only (larger studies included all NTDs), and we were able to further restrict our analyses to only myelomeningocele in sensitivity analyses.

## Conclusions

Environmental arsenic exposure may reduce the protective effects of folic acid supplementation on spina bifida risk. Our findings raise important questions about the risk of spina bifida in arsenic-endemic areas and the effectiveness of folic acid supplements alone as the strategy to prevent spina bifida in high-arsenic areas of the world. At minimum, more surveillance for spina bifida is needed in high arsenic areas such as Bangladesh. Additional preventive measures, such as folic acid fortification of the food supply and reduction of arsenic exposure, may be needed to optimize spina bifida prevention in areas with high arsenic exposures.

### Electronic supplementary material

Below is the link to the electronic supplementary material.

Supplementary Material 1

## Data Availability

No datasets were generated or analysed during the current study.

## Abbreviations

BCH: Boston Children’s Hospital
CSF: Cerebrospinal fluid
CI: Confidence interval
DSH: Dhaka Shishu Hospital
GF-AAS: Graphite furnace atomic absorption spectrometry
ICP-MS: Inductively coupled plasma mass spectrometry
LOD: Limit of detection
NINS&H: National Institute of Neurosciences & Hospital
NTD: Neural tube defect
OR: Odds ratio
SAM: S-adenosylmethionine
