# Supplementary material for: Arsenic modifies the effect of folic acid in spina bifida prevention, a large hospital-based case-control study in Bangladesh
Source: Environ Health. 2024 Jun 3;23:51. doi: 10.1186/s12940-024-01091-1 (PMC11145859; doi:10.1186/s12940-024-01091-1)
Supplement: Supplementary file 1 — Supplementary Material 1 [file 12940_2024_1091_MOESM1_ESM.docx]

**Supplemental Figure 1**. *Association between prenatal folic acid use and myelomeningocele risk, stratified by mothers’ toenail arsenic concentration.*


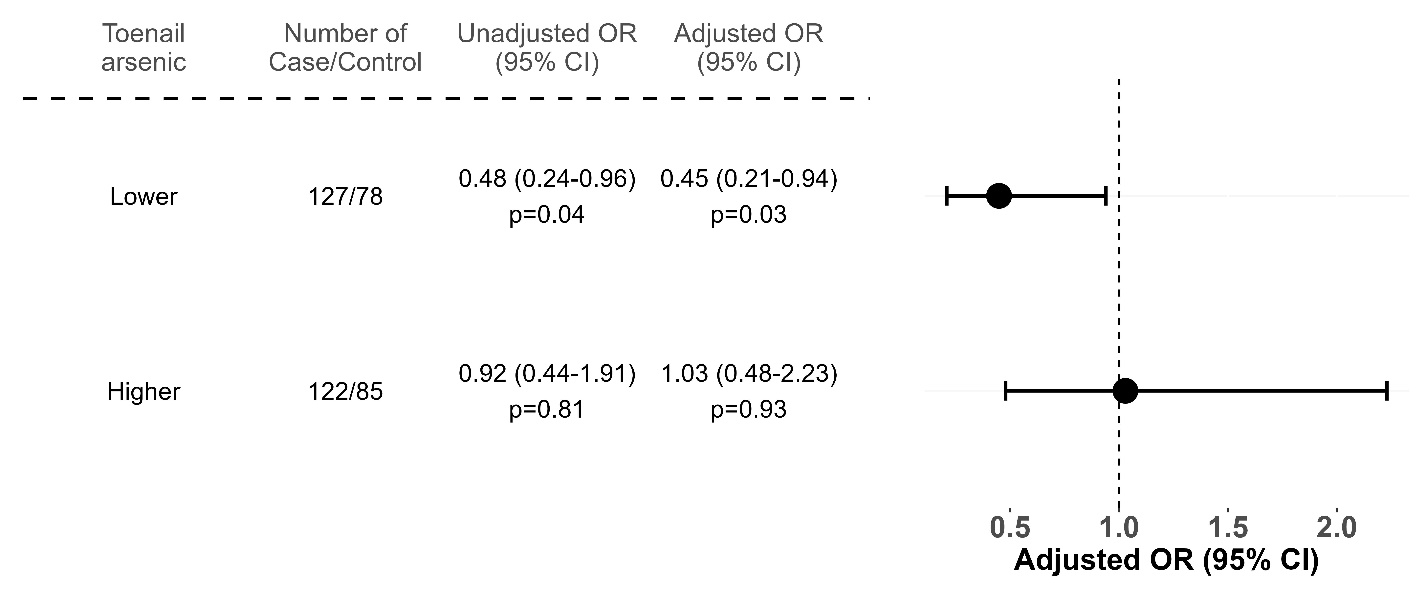


Adjusted for mothers’ age (years), place of birth (hospital, clinic, or homes), and secondhand smoke exposure. The cutoff for mothers’ toenail arsenic concentrations was defined by the median of study population (0.46 µg/g toenail). Abbreviations: CI, confidence interval; OR, odds ratio.

**Supplemental Figure 2**. *Association between prenatal folic acid use and spina bifida risk, stratified by water arsenic concentration.*


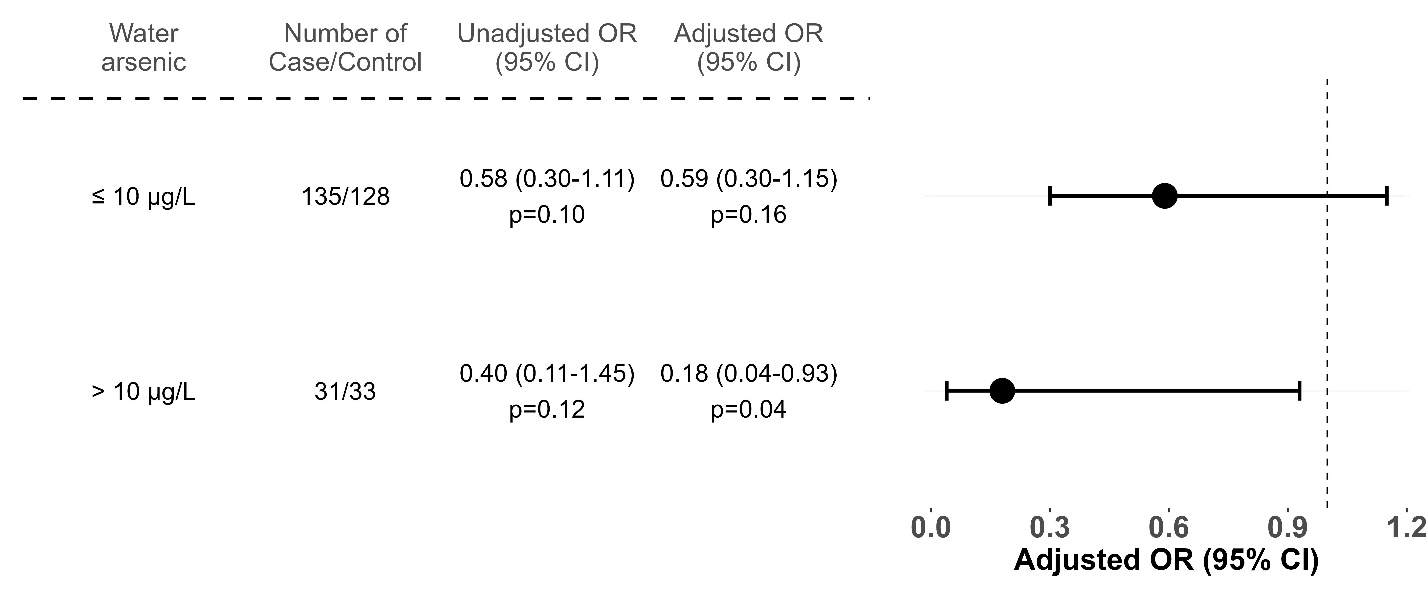


Adjusted for mothers’ age (years), place of birth (hospital, clinic, or homes), and secondhand smoke exposure. The cutoff for water arsenic concentrations was the WHO standard for drinking water (10 µg/L). Abbreviations: CI, confidence interval; OR, odds ratio; WHO, World Health Organization.

**Supplemental Table 1.** *Eligibility criteria for cases and controls.*

| ***Case*** |
| --- |
| - Diagnosed with myelomeningocele or meningocele. - Aged one year or younger. - Received medical care from the National Institute of Neurosciences & Hospital (NINS&H) or was referred to NINS&H from an outside hospital or clinic. - The family can identify the mother's drinking water source at the time she learned she was pregnant. - A child can be recruited as a case even if they have already undergone surgery for spina bifida. |
| ***Control*** |
| - The family can identify the mother’s drinking source at the time she learned she was pregnant. - Received medical care from NINS&H or nearby Dhaka Shishu Hospital (DSH). - Age (± 6 months) as a case. - No diagnosis of cancer. - A child cannot be recruited if their mother used the same water source during pregnancy as the mother of a case. |

1. We stopped control enrollment during COVID-19 pandemics in Bangladesh.

| **Supplemental Table 2.** *Diagnoses of the controls.* | |
| --- | --- |
| **Diagnosis** | **No. (%)** |
| Epilepsy | 73 (44.8) |
| Hypoxic ischemic encephalopathy | 32 (19.6) |
| Subdural Effusion | 18 (11.0) |
| Craniosynostosis | 16 (9.8) |
| Trauma | 8 (4.9) |
| Arachnoid cyst | 6 (3.7) |
| Others | 5 (3.1) |
| Epidermoid cyst | 4 (2.5) |
| Vascular disease | 1 (0.6) |
| **Total** | 163 |

**Supplemental Table 3.** *Associations between water and mother’s toenail arsenic concentrations and spina bifida risk.*

|  | **Unadjusted model** | **Adjusted model ^a^** |
| --- | --- | --- |
|  | OR (95% CI, p-value) | OR (95% CI, p-value) |
| ***Water arsenic*** |  |  |
| Per unit change (μg/L) | 1.00 (0.99-1.00, p=0.30) | 1.00 (0.99-1.00, p=0.37) |
| > 10 μg/L | 0.89 (0.52-1.54, p=0.68) | 0.87 (0.50-1.52, p=0.63) |
| ***Toenail arsenic*** |  |  |
| Per unit change (µg/g toenail) | 0.97 (0.87-1.08, p=0.58) | 0.97 (0.87-1.09, p=0.61) |
| > median (0.46 µg/g toenail) | 0.87 (0.59-1.27, p=0.47) | 0.85 (0.57-1.25, p=0.39) |
| 1. Adjusted for mothers’ age (years), place of birth (homebirth, clinics, or hospitals), and secondhand smoke exposure. | | |
| Abbreviations: CI, confidence interval; OR, odds ratio. | | |
